# Agent-based dynamic knowledge representation of *Pseudomonas aeruginosa *virulence activation in the stressed gut: Towards characterizing host-pathogen interactions in gut-derived sepsis

**DOI:** 10.1186/1742-4682-8-33

**Published:** 2011-09-19

**Authors:** John B Seal, John C Alverdy, Olga Zaborina, Gary An

**Affiliations:** 1 Department of Surgery, University of Chicago, 5841 South Maryland Ave. MC 5031, Chicago, IL 60637, USA

## Abstract

**Background:**

There is a growing realization that alterations in host-pathogen interactions (HPI) can generate disease phenotypes without pathogen invasion. The gut represents a prime region where such HPI can arise and manifest. Under normal conditions intestinal microbial communities maintain a stable, mutually beneficial ecosystem. However, host stress can lead to changes in environmental conditions that shift the nature of the host-microbe dialogue, resulting in escalation of virulence expression, immune activation and ultimately systemic disease. Effective modulation of these dynamics requires the ability to characterize the complexity of the HPI, and dynamic computational modeling can aid in this task. Agent-based modeling is a computational method that is suited to representing spatially diverse, dynamical systems. We propose that dynamic knowledge representation of gut HPI with agent-based modeling will aid in the investigation of the pathogenesis of gut-derived sepsis.

**Methodology/Principal Findings:**

An agent-based model (ABM) of virulence regulation in *Pseudomonas aeruginosa *was developed by translating bacterial and host cell sense-and-response mechanisms into behavioral rules for computational agents and integrated into a virtual environment representing the host-microbe interface in the gut. The resulting gut milieu ABM (GMABM) was used to: 1) investigate a potential clinically relevant laboratory experimental condition not yet developed - i.e. non-lethal transient segmental intestinal ischemia, 2) examine the sufficiency of existing hypotheses to explain experimental data - i.e. lethality in a model of major surgical insult and stress, and 3) produce behavior to potentially guide future experimental design - i.e. suggested sample points for a potential laboratory model of non-lethal transient intestinal ischemia. Furthermore, hypotheses were generated to explain certain discrepancies between the behaviors of the GMABM and biological experiments, and new investigatory avenues proposed to test those hypotheses.

**Conclusions/Significance:**

Agent-based modeling can account for the spatio-temporal dynamics of an HPI, and, even when carried out with a relatively high degree of abstraction, can be useful in the investigation of system-level consequences of putative mechanisms operating at the individual agent level. We suggest that an integrated and iterative heuristic relationship between computational modeling and more traditional laboratory and clinical investigations, with a focus on identifying useful and sufficient degrees of abstraction, will enhance the efficiency and translational productivity of biomedical research.

## Introduction

### Dynamic host-microbe interactions in the gut: A new paradigm for microbe-associated disease

The understanding of how microbes cause disease has evolved dramatically since the introduction of Koch's postulates and development of germ theory over a century ago. Humans represent "below the skin" ecosystems, supporting vast and diverse intestinal communities of microbial species that serve important roles in digestion, metabolism and development. There is an increasing recognition of the importance and influence of the gut microbiome in various disease states [1-3]. The host-microbe dialogue can be transformed by changes in the constituent species or genetic background of colonizing flora, impairment of host defenses, or physiologic perturbations brought about by host stress [4-6]. Recent evidence suggests that potentially pathogenic microbes undergo virulent transformation during conditions of host stress [7-20]. Physiologic changes associated with critical illness, coupled with consequent modern medical therapies, can lead to escalation of virulence expression, immune activation and ultimately systemic inflammatory dysregulation [7,21]. Given the scale and anatomic differentiation of the interactive surface of the gut there will be considerable regional heterogeneity in terms of bacterial species and local host factors. Therefore, it is reasonable to characterize the gut ecosystem as a series of microenvironments where regional differences in host conditions and bacterial populations can lead to divergent ecological trajectories.

Host-pathogen interactions (HPI) consist of a series of mechanistic molecular-based processes where microbial and host cells sense, respond to and influence their local environments. While mechanisms for this phenomenon have been described for many pathogens, we use the virulence activation in *Pseudomonas aeruginosa *in response to a stressed gut milieu, and the effect of thusly activated *P. aeruginosa *on that milieu as our model reference system. *P. aeruginosa *is a gram negative bacillus that is one of the most clinically significant microbes in hospital settings, with a high degree of morbidity and mortality associated with its presence [22]. *P. aeruginosa *virulence expression has been identified as responding to local environmental cues, many of which are host tissue factors released in response to physiologic stress, such as tissue ischemia [9], immune activation [8], phosphate depletion [23-25] and endogenous opioid response [26]. Each of these conditions corresponds to commonly observed clinical responses in critically-ill, stressed patients, and in many clinical scenarios several, if not all, of these host responses occur contemporaneously as a part of a global physiologic stress state. These alterations in the baseline host physiological state may disturb the balance of the baseline, non-pathologic HPI, and therefore may represent potential targets for translational research directed at preventing a pathogenic shift in the HPI. We focus on representing the mechanisms and consequences of *P. aeruginosa *virulence activation in the gut of a stressed host as an example of how HPI associated with clinical disease can be investigated through an iterative integration between traditional experimental workflow and dynamic computational modeling. There are four thematic goals in this process:

1. The integration and dynamic representation of mechanistic knowledge of the complex processes of *P. aeruginosa *virulence activation in the stressed gut. This is primarily reflected in ABM development and initial implementation.

2. To use that dynamic representation as a means of knowledge visualization and conceptual model verification: i.e. can the instantiation of the mechanistic hypothesis in achieved in Goal 1 be made to behave in a plausible and recognizable fashion? This is primarily accomplished in the initial model-testing phase of development (cross-model validation).

3. To use the resulting GMABM as an *in silico *adjunct to examine experimental conditions not currently explored using traditional experimental methods. This is primarily manifest in the design and execution of simulation experiments.

4. Formulate new hypotheses arising from observed discrepancies between the ABM and real-world observations and suggest how new experiments might be performed to test these new hypotheses. This process takes place during the interpretation and analysis of the simulation experiments.

These goals represent a sequential process that mirrors the general scientific method; we aim to demonstrate that the execution of that process within the context of developing and using a computational model can enhance the standard scientific workflow.

### *In silico *dynamic knowledge representation of the HPI

The spatio-temporal biocomplexity of the host-microbe relationship has come into focus as a key aspect of understanding the pathogenesis of clinical infections [27,28]. While molecular techniques for describing mechanistic details of microbial and host physiology have yielded tremendous advances in characterizing mediators and pathways, reassembling that knowledge in a useful and practical context that effectively represents the behavior of this complex biological system remains a formidable challenge. Techniques from systems biology can facilitate the integration, visualization and manipulation of mechanistic knowledge and improve translational efforts [29-31], but there is a clear need to be able to expand beyond the level of individual cells and characterize the behavior of cellular populations [32,33].

Agent-based modeling represents one technique that offers specific advantages for modeling spatially diverse, dynamic, multi-factorial systems, such as HPI in the gut [31,34,35]. Agent-based models (ABMs) are composed of virtual environments populated with objects (agents) that execute behaviors based on programmed rules that govern interactions with the local environment and other agents. The behavioral rules for an agent can range in complexity from a series of Boolean conditional statements to highly sophisticated mathematical models and decision algorithms, giving ABMs to capacity to potentially incorporate multiple levels of mechanistic resolution and detail. During execution of an ABM individual agent behaviors can vary based on differing local conditions, and, in aggregate, produce population-level dynamics that represent the dynamics of the system as a whole. Agent-based modeling has been used to dynamically represent aspects of complex biological processes including inflammation [29,36-41], cancer [42-45], infectious diseases [46-50] and wound healing [51,52]. There is also a growing recognition of the importance of spatial heterogeneity and population effects in ecology [53-55], immunology [48,49,56-58] and epidemiology [59,60]. By capturing the transition from individual agent behavior to the behavior of populations of agents, ABMs are able to produce non-intuitive behavioral patterns that may only manifest at the system-level. Examples of this type of system-level behavior include phase transitions in physical systems [61], flocking/schooling behavior in birds [62], fish [63] and other ecological systems [64] and quorum sensing in bacteria [65,66].

Being able to capture this type of system-level phenomena is of critical importance in the investigation of biological systems, since there are several levels of organization between the level of mechanism targeted for putative control (often gene/molecule) and the clinical relevance/implications of that intervention (whole organism). Each of these levels of organization, extending from gene to molecule to cell to tissue to organ to organism, represents a potential epistemological boundary where inferred consequences at a higher level of organization cannot be assumed from identified mechanisms at a lower level. These boundaries challenge the fidelity of the *modeling relation *between an experimental model (be it a biological lab system or a computational simulation) and the biological referent, where the *modeling relation *is defined as the mapping of the generative processes and generated outputs between the model and its referent [34,67]. Agent-based modeling used for dynamic biomedical knowledge representation is a means of making the modeling relation more explicit. Executing an ABM also evaluates the dynamic consequences of a particular mechanistic hypothesis by extending the experimental context in which those mechanisms are executed, i.e. to a higher level of biological organization. Dynamic knowledge representation aims to bridge gaps between the context in which mechanisms are identified (i.e. pathway information identified through in vitro experiments) and the multiple ascending scales/contexts present during the translation of that knowledge into the clinical/organism level (i.e. cell => tissue => organ => organism). We assert that one of the primary modeling relation transitions in the study of biological systems occurs in the extrapolation of single cell behavior into cellular population behavior at the tissue level. With this in mind, we have chosen the cell-as-agent resolution level as a means of bridging the intra-cellular molecular knowledge derived from *in vitro *experimental investigations to the population-level, space-incorporating, tissue and organ level context necessary to represent clinically relevant behavioral dynamics.

### Establishing Plausibility: The benefits of detailed, selectively qualitative dynamic knowledge representation

Related to the issue of explicit representation of the modeling relation in the study of biological systems is the question of what constitutes an appropriate level of model representation and detail? The scope and scale of a modeling project is intimately tied to and informed by its use. This is often termed establishing the *experimental frame *[68,69]. Given the limits and incompleteness of biological knowledge, a pragmatic goal of biomedical modeling and simulation is to aid in the discovery and evaluation of potential plausible mechanisms. When operating within this discovery-oriented experimental frame the first step in the evaluation of a hypothesis is determining its *face validity*, and thus its plausibility. *Face validity *is defined as the ability of a particular simulation to behave in a realistic, reasonable and believable manner, and represents the first tier in a validation sequence used for engineering simulations [68-70]. Often the criteria for determining face validity are qualitative by nature: i.e. "Does this behavior look right?" For example, such criteria might be that model behavior approximate the behavior of the referent in terms of relative magnitude and timeline, and that actual and predicted changes in model behavior occur in the same general direction as seen in the referent. While admittedly a low bar in terms of assessment, the standard of face validity is a useful and arguably necessary step while engaged in the "discovery" phase of science; the behavior of putative hypotheses reasonably should at least pass this test in order to be eligible for more rigorous testing [70]. Establishing face validity can involve cross-model validation: the comparison of the output of the computational model to a specific real world referent, which may itself be a reduced experimental model of a more complex biological subject. This process includes trying to "coerce" the computational model (generally through parameter manipulation) to reproduce data from the referent; the inability to do so within the bounds of plausible manipulation (for example, if cells are required to move at rates not compatible with the implementation of their other functions in order to produce a desired model output) suggests that the underlying hypothesis structure is incorrect. Conversely, if the computational model is able to generate output acceptably matched to data from its referent, it is considered to be plausible and is subjected to further use and testing.

### ABM of HPI in the gut milieu

The use of agent-based modeling for this type of knowledge representation has been previously described in the biomedical arena [36,71-73], and represents our strategy for the development of an ABM concerning *P. aeruginosa *virulence activation in the stressed gut. We aim to represent virulence-associated signal transduction and gene regulatory processes identified in *P. aeruginosa *with a relatively high degree of component detail, but abstracted in terms of the actual biochemical kinetics. Rate constants for classes of biochemical events are assumed to operate within qualitative orders of magnitude, and therefore, highly-abstracted representation of biochemical kinetics, as either Boolean, logic-based or algebraic statements, can be of sufficient descriptiveness to produce ABM behavior that pattern-matches those seen in the experimental data [73-76]. We note that when using this approach the relationship between the components (and their respective mechanisms) is of critical importance [34,67]. Our emphasis on "selectively qualitative" can be considered a means of relational representation and grounding, as we focus on representing the relationships between the modeled components to produce recognizable and plausible behaviors.

The current ABM represents an initial, relatively abstract example of dynamic knowledge representation of the gut HPI, and in the future the modular nature of ABM will allow graduated addition of agents and variables (such as inflammatory cells, goblet cells, sub-epithelial tissue architecture and vascular system), as well as more complex rules for individual agents (such as mathematical models of signal transduction or genome-scale metabolic models), to produce higher resolution models of the gut HPI. However, we suggest that dynamic knowledge representation using even relatively abstract ABMs can play a useful role in the current scientific process.

## Methods

### Overview

We developed a series of ABMs of virulence regulation in *P. aeruginosa *using Netlogo, an agent-based modeling software toolkit [77]. The rules for agents representing *P. aeruginosa *were developed using a series of modular submodels, each submodel focusing on a particular set of *in vitro *experiments examining one particular activation pathway by host-derived stress signals: immune-activation, mediated through the molecule interferon-γ (IFN-γ) [8], ischemia, manifest as reduction in blood flow and oxygen availability, and reflected in the production of adenosine [9], endogenous opioids, manifest as dynorphin [26], and phosphate depletion, seen concurrent with major surgical stress [23-25]. The rules for agents representing gut epithelial cells were abstracted from previously published ABMs involving tight junction metabolism and inflammatory response in gut epithelial cells [36]. Submodels were cross-model validated to data from their corresponding experimental referents, and then integrated into an aggregated ABM that included additional organ-level variables (mucus, commensal flora, nutrients and soluble host factors) to simulate an *in vivo *gut environment of a stressed host. We term this integrated ABM the gut milieu agent based model (GMABM). A text file of the code for the GMABM can be seen in Additional File 1 while the Netlogo model can be downloaded from http://bionetgen.org/SCAI-wiki/index.php/Main_Page.

The process of constructing the GMABM, which we treat as an analog to *in vivo *experimental models, is similar to the knowledge transfer associated with "wet lab" progression from *in vitro *models to more complex animal models, with the added benefit of having explicit transparency in terms of represented mechanisms. Conditions of systemic host stress were then simulated to observe interactions between Pseudomonas agents and the gut barrier manifest as alterations in population characteristics, spatial distribution of effects, and aggregate system-level variables. The results of these simulations were compared with animal models (*in vivo *referents) to evaluate the plausibility of interactions and to identify knowledge gaps when outcomes were divergent. It should be noted that the agent-rule structures were not changed in the process of submodel integration other than at necessary points of submodel intersection (i.e. shared components).

In an effort the move towards standardization of ABM development and analysis, Grimm, et al. have described the Overview, Design Concepts, Details (ODD) protocol to describe the construction and use of an ABM [78]. This protocol was initially developed with ecological modeling in mind, though its use has been expanded to other applications of agent-based modeling [48,78]. We have used a modified version of the ODD protocol as the organizational structure of this Methods section.

### Design Concepts: Utilizing a bacteriocentric perspective

Existing published ABMs of HPIs during infection have a distinctly immunocentric focus with simplification of the spatial and temporal aspects of phenotypic expression of pathogens [57,79-81]. Alternatively, the bacteriocentric organization of the GMABM emphasized the mechanisms of microbe virulence activation and represents host functions primarily as modifiers of the mucus milieu by secretion of signaling molecules and depletion of resources. While in biological referents the host response to pathogens is quite involved, especially with respect to adaptive and innate immune components, representation of host defenses was limited in the GMABM to basic barrier functions associated with gut epithelial cells and an abstracted immune response in order to focus the GMABM on virulence regulation in *P. aeruginosa*. We recognize the potential limitations of this approach, but given the focus of prior investigations we believe that we can provide a novel scientific contribution through our bacteriocentric focus.

### Entities, State Variables and Scales

**Figure 1:**
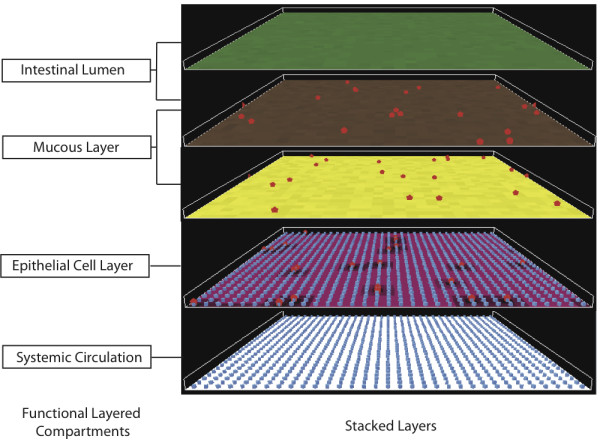
**Architecture and topology of the ABM**. The ABM simulates the 3-dimensional relationships of the gut-luminal interface by utilizing "stacked" data layers, each one representing a two-dimensional aspect of the gut-microbial interaction environment. It should be noted that the "stacking" occurs only in a virtual sense. This approach is akin to that used in geographical information systems (GIS) [102]. Representative layers depicted include luminal phosphate concentration (green patches), endogenous gut flora population (brown patches), mucous barrier (yellow patches), and epithelial cell tight junctions (violet patches). Agents interact within and between data layers as depicted by Pseudomonas agents (red pentagons) in the mucous and epithelial layers and epithelial cell agents (blue squares) in the epithelial cell layer and interface with the systemic circulation. Simulation world data is passed from one data-layer to the next based on encoded rules in the ABM. Run-time visualization of model layers or variables can be modified at the user interface with application of filters for specific variables to be displayed in the 2-dimensional graphical interface (see Figure 2).

**Figure 2:**
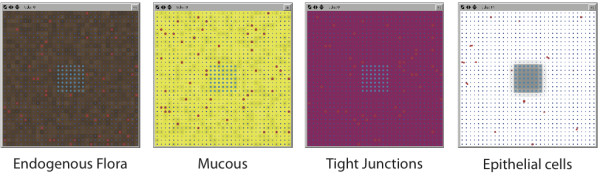
**Screenshots of different backgrounds representing data layers**. Representative patch backgrounds depicting endogenous gut flora population (brown patches), mucous barrier (yellow patches), epithelial cell tight junctions (violet patches) and epithelial cells (blue GECs on white background). Shading of background color reflects quantitative changes in specific variables (e.g. mucous, endogenous flora, tight junctions). Pseudomonas agents (red pentagons) move to survey microenvironments while epithelial cells (blue squares) modify local conditions in response to host stress. This feature of the ABM aids in initial code development to visually identify encoded behaviors, provides visual reinforcement of expected model behavior and facilitates the use of visual intuition to identify patterns and behaviors that might not be evident in purely tabular data output.

The agent level of the ABM is the cellular level, representing individual *P. aeruginosa *bacteria ("Pseudomonas agents") and gut epithelial cells ("GEC agents"). The spatial configuration of the ABM is a 2-dimensional square grid with the 3^rd ^dimension represented as 4 overlying data layers: the intestinal lumen, the gut mucus layer, the gut epithelial layer and the systemic circulation (see Figure 1 and 2). The grid is toroidal, as to avoid edge effects. The GMABM is abstracted with one grid space ("patch") approximating one GEC agent. GEC agents reside in the gut epithelial layer. At baseline, Pseudomonas agents reside in the gut mucus layer; if the mucus is depleted then they can directly interact with the GEC agents. There is an arbitrary limit of 20 Pseudomonas agents per patch, representing the maximal number of Pseudomonas agents that can reside on the discretized space represented by a single patch and are treated as a well-mixed population within the spatial resolution of the patch. Patch variables include extra-cellular molecules and populations of commensal bacteria; extracellular molecules are specified with tags associating them with their model layer location: lumen, mucus, epithelial and circulatory. There are three patch variables not generated by cellular agents (Pseudomonas or epithelial): phosphate, mucus and commensal bacteria. The first two have a random value (normal distribution) within a range: phosphate between 0 and 99 where the upper value can be varied as an experimental condition, and mucus between 90 and 100 not varying unless degraded by activated Pseudomonas agents. The variability of the values is meant to reflect the heterogeneous nature of the gut environment. Commensal bacteria are modeled as an aggregate population variable within the gut mucus layer rather than individual agents due to their relatively passive role in the GMABM (see below in the Submodel section). The state variables for the Pseudomonas agents and the GECs represent molecular level components internal to the cells: receptors, signaling factors, gene transcription factors, genes and structural molecules. The molecular pathways are represented qualitatively, thus the corresponding variables are unit-less, but with a considerable degree of component detail, consistent with our previously described method of detailed, selectively qualitative modeling [36,71-73]. This approach consists of relatively detailed component representation (i.e. including specific enzymes, molecular species and genes) with qualitative representation of biochemical kinetics using a fuzzy Boolean logic-based rule construction. Molecular interaction rules are expressed as conditional statements of the form:

if Ligand A is present (or above some threshold), then bind to and activate Receptor B

if Receptor B is activated, then increase Signal Transduction Enzyme C by 1

And so on...

**Figure 3:**
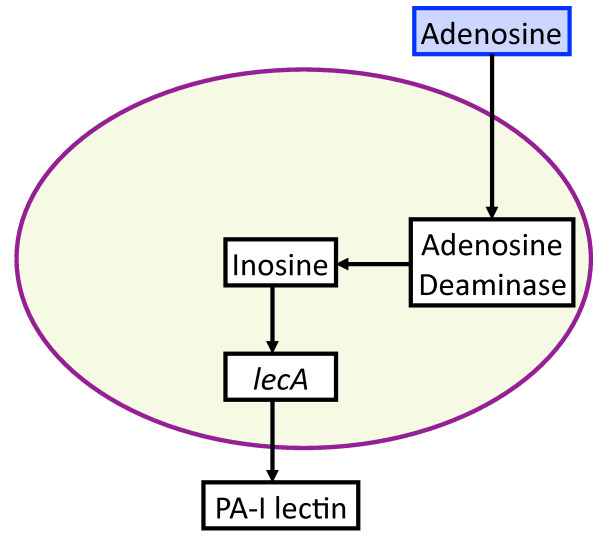
**Schematic of *P. aeruginosa *virulence activation pathway due to adenosine, a host product of ischemia/reperfusion**. Intestinal ischemia and reperfusion leads to the production of HIF-1α, which induces the release of adenosine into the intestinal lumen. Adenosine is transported into the bacterial where it is converted to inosine by adenosine deaminase. Inosine induces the expression of the coding region lecA, which is transcribed and translated into the protein PA-I lectin, which is secreted into the intestinal lumen and causes epithelial barrier dysfunction. All the above molecular components are represented by state variables in the GMABM, and the directional arrows indicate the presence of state transition rules.

**Figure 4:**
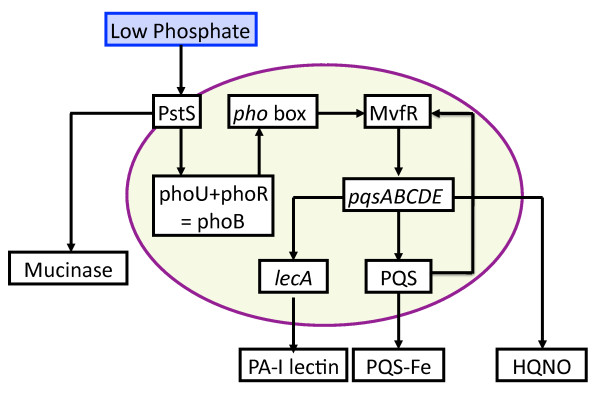
**Schematic of *P. aeruginosa *virulence activation pathways due to bacterial sensing of low phosphate**. Low phosphate in the mucous layer of the intestine is sensed by *P. aeruginosa *through PstS protein. This activation of PstS results in changes in the Pst-PhoU-PhoR complex, leading to histidine kinase PhoR phosphorylation and activation of the transcriptional regulator PhoB that then binds to the *pho *box gene sequence that controls hundreds of genes including those encoding main regulators of quorum sensing, such as MvfR. MvfR is a transcriptional regulator that acts upstream of the operon *pqsABCDE*, which codes for, among other things, the enzymes that lead to the production of PQS, a quorum sensing compound, and the bactericidal compound HQNO. PQS serves three additional functions: 1) activates *lecA*, which leads to the production of PAI-lectin, 2) is secreted to bind to free iron (Fe), and 3) feeds back to enhance the binding of MvfR to the promoter sequence upstream of *pqsABCDE*. All the above molecular components are represented by state variables in the GMABM, and the directional arrows indicate the presence of state transition rules.

**Figure 5:**
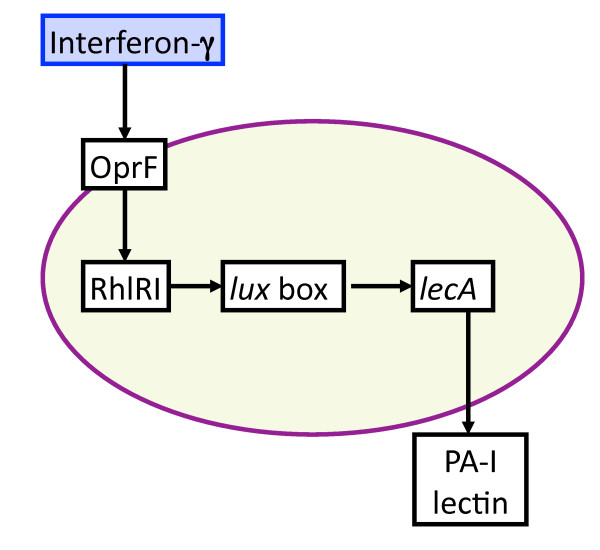
**Schematic of *P. aeruginosa *virulence activation pathways due to interferon-γ, a product of host inflammation**. Host cells subject to inflammation secrete the cytokine interferon-γ (IFN-γ). IFN-γ binds to outer membrane porin OprF on *P. aeruginosa*. Bound OprF activates RhlI, a N-(butanoyl)-L-homoserine lactone synthetase in the quorum sensing system, which in turn is required for PA-I lectin production. All the above molecular components are represented by state variables in the GMABM, and the directional arrows indicate the presence of state transition rules.

**Figure 6:**
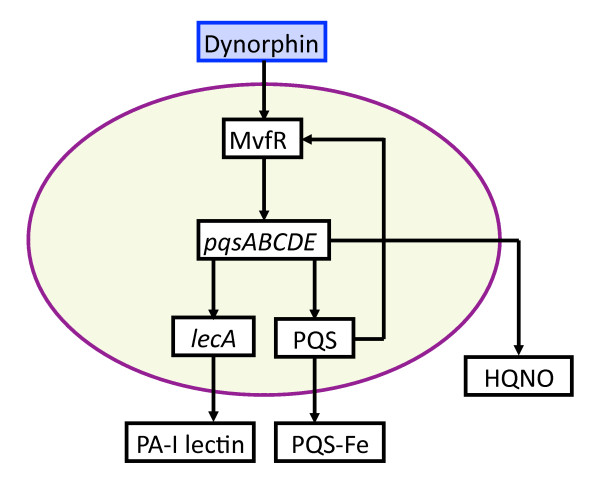
**Schematic of *P. aeruginosa *virulence activation pathways due to bacterial sensing of endogenous opioids, a product of host stress**. Endogenous opioids are release by host tissues during systemic stress. Dynorphin, a synthetic agonist used to study opioid receptors, activates the transcriptional regulator MvfR, and leads to the expression of its regulated operon *pqsABCDE *and subsequent downstream products production of HQNO, and PQS, as noted above in the low phosphate signaling pathways (Figure 4). All the above molecular components are represented by state variables in the GMABM, and the directional arrows indicate the presence of state transition rules.

**Figure 7:**
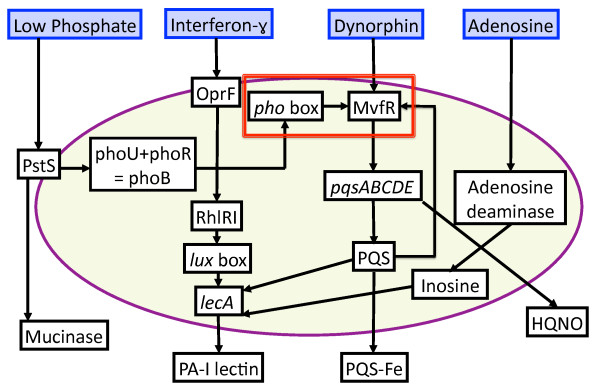
**Schematic of aggregated *P. aeruginosa *virulence activation pathways associated with host systemic surgical stress**. A summary of the four virulence pathways depicted in Figures 3, 4, 5 and 6 is presented in aggregated form. Note the points of convergence and intersections among the different pathways, particularly in terms of downstream effects, suggesting highly conserved and advantageous functions for the virulence outputs of *P. aeruginosa*. Also note the putative link between low phosphate sensing and opioid sensing reflected by the association between *pho *box and MvfR (seen in the box outlined in Red). MvfR is clearly an important control point in the phenotypic switching between non-virulent and virulent states, and represents a target for future investigation.

For a comprehensive list of entities and state variables included in the GMABM see Table 1; the corresponding biological description of these entities can be seen in Table 2. Figures 3, 4, 5, 6 and 7 are schematic directed graphs of the various virulence pathways implemented in the GMABM Pseudomonas agents. In the ABM each node-edge-node relationship displayed in the schematic is represented by fuzzy Boolean rules in the general format noted above. The code of the GMABM can be seen in Additional File 1.

**Table 1:** ABM Agent Types, Model Variables and Manifestation in ABM Rules

Agents and Variables	Rules
***Epithelial cell agents***	***One agent per patch, fixed (Blue Squares)***

i-Interferon-γ	Intracellular production of interferon-ϒ released during inflammation

i-Dynorphin*	Intracellular production of dynorphin, released during ischemia/reperfusion, dynorphin expression enhanced by factor of 3 when Pseudomonas agent present

i-Adenosine	Intracellular production of adenosine, released during ischemia

HIF-α	Intracellular signal for adenosine production during ischemia

TJ-level	Intracellular production of tight junction proteins, turnover 90 minutes

***Pseudomonas agents***	*Random distribution, heading, and movement (Red Pentagons)*

i-Dynorphin*	Uptake of extracellular dynorphin and activator of mvfr

oprF*	Membrane-bound receptor, activation proportional to [interferon-ϒ]

RhlRI*	Conserved quorum-sensing molecule, regulated by oprF

*Lux*box*	Response element upstream of lecA

i-adenosine	Uptake of extracellular adenosine

Adenosine-deaminase*	Converts adenosine to inosine

Inosine*	Activates *lecA*

PstS*	Membrane-associated protein, activation proportional to [Pi]

PhoR*	Intermediate phosphate signaling molecule

PhoB*	Intermediate phosphate signaling molecule, binds to phobox

*pho *box*	Response element upstream of *mvfr*

lecA*	Gene for PA-I lectin expression, activated by inosine, PQS, luxbox

i-PA-I-lectin	Intracellular production of PA-I lectin, causes binding to epithelial cells

Mvfr*	Multiple virulence factor, upstream promoter for quorum sensing virulence

TNA*	Downstream to mvfr (See *mvfr *box in Table 2).

pqsABCDE*	Downstream to TNA

i-HQNO*	Intracellular QS intermediate molecule, toxic to *Lactobacillus *spp.

i-PQS*	Intracellular QS intermediate molecule, activates lecA, form epithelial toxin

Grow-colony	Proxy for growth signal when resources (mucous layer) > endogenous flora

Quorum-sense	Recognizes quorum based on concentration of quorum-signal

***Patch Variables***	

Mucous	Initial value between 90 and 100 per patch (normal distribution), remains constant and determined carrying-capacity for gut environment (proxy for food, space, shear clearance)

Phosphate	Initial concentration random value in normal distribution between 0 and 99 (arbitrary units), where the upper value is controlled through the user interface as an experimental condition

Endogenous flora	Initial population at maximum carrying capacity, growth impaired by HQNO

HQNO*	Produced by Pseduomonas agents, a toxin that impairs growth of endogenous flora, decreases competition allows for population growth

PA-I lectin*	Produced by Pseudomonas agents, a toxin that causes epithelial barrier dysfunction

Quorum-signal	Produced by Pseudomonas agents, an intercellular communication molecule by which Pseudomonas agents sense Pseudomonas density

This table presents a list of the agent classes representing cellular, bacterial and environmental types, variables of those types corresponding to identified mediators and compounds, and the rule-sets for behavior involving those compounds as instantiated in the ABM. It should be noted that the list of rules reflects the programming code semantics for the biological mechanisms of the simulated compounds. For a more detailed biological description of selected compounds (noted by an asterisk "*") readers are directed to Table 2.

**Table 2:** Biological Description of Selected Simulation Rules and Variables

Compound	Biological Description
Dynorphin	Class of opioid peptides, activator of MvfR

OprF	Outer membrane protein, binds INF-γ to enhance virulence

RhlRI	Quorum sensing subsystem composed of RhlI, the C4-HSL (N-butyrylhomoserine lactone) autoinducer synthase and RhlR transcriptional regulator, activates as a consequence of binding INF-γ to OprF

*lux *box	DNA sequence with dyad symmetry located in the promoter regions of many quorum-sensing-controlled genes including *lecA*. Functions as binding site for quorum sensing transcriptional regulators RhlR and LasR.

Adenosine-deaminase	Converts adenosine to inosine

Inosine	Activates *lecA *expression

PstS	Phosphate-binding protein, induced by phosphate limitation

PhoR	Two-component (PhoR/PhoB) sensor kinase, activated during phosphate limitation as a consequence of PstS expression.

PhoB	Two-component (PhoR/PhoB) transcriptional regulator for phosphate regulon genes. Phosphorylation of PhoB by PhoR enhances its binding activity to *pho *box.

*pho *box	DNA conserved sequence located in promoter region of phosphate regulon genes, including *mvfR*.

*lecA*	Gene encoding PA-I lectin, the expression is regulated by quorum sensing. Exposure of *P. aeruginosa *to epithelial cell agents adenosine, opioid, and INF-γ induces the expression of *lecA*.

MvfR	*P. aeruginosa *LysR-type transcriptional regulator, modulates the expression of multiple quorum sensing (QS)-regulated virulence factors, regulates the biosynthesis of 4-hydroxy-2-alkylquinolines (HAQs) including HQNO and PQS.

*mvfR *box (corresponds to TNA in Table 1)	DNA consensus palindromic sequence T-[N]_11_-A with a dyad symmetry located in promoter region of MvfR-regulated genes including *pqsABCDE*.

*pqsABCDE*	Operon regulated by MvfR, encodes proteins required for the biosynthesis of HQNO and HHQ, a precursor of PQS. HHQ and PQS potentiate MvfR binding to *mvfR *box upstream of *pqsABCDE *forming feedback loop regulation.

HQNO	4-hydroxy-2-heptylquinoline-N-oxide, the *P. aeruginosa *exoproduct regulated by QS, suppresses the growth of many gram-positive bacteria including *Lactobacillus *spp., mediates protection of *Staphylococcus aureus *against aminoglycosides antibiotics.

PQS	2-heptyl-3-hydroxy-4(1 H)-quinolone, the *P. aeruginosa *exoproduct regulated by QS, plays multifunctional role in quorum sensing including intra-cellular and inter-cellular signaling. Shapes the population structure of *Pseudomonas *and response to and survival in hostile environmental conditions. Induces apoptosis in mammalian cells.

PA-I lectin	Pseudomonas toxin causes potent epithelial barrier dysfunction

This table presents a more detailed biological description of selected compounds within the ABM (items with an asterisk "*" from Table 1) that are specifically related to gut host-microbial crosstalk and virulence activation. Readers are encouraged to examine Tables 1 and 2 to see how biological descriptions are converted to ABM rules.

### Collectives and Observations

The set of observables for the GMABM is informed and determined by the type of data generate by the biological referents, be they *in vitro *or *in vivo *models. The scalar metric output of the GMABM for cross-model validation and simulation experiments are population metrics that represent aggregated output from the individual agents in the GMABM. These scalar metrics correspond to global levels of mediators (measured from the GMABM as a whole) and cell populations, either in total for an agent class or a specific subpopulation. This data can be seen in the outputs of the cross-model validations and simulation experiments. In addition to these scalar metrics, visual patterns of the simulation world observable through Netlogo's graphical user interface. While not quantitative information, the visualized behavior of the GMABM provides a qualitative means of evaluating the plausibility of the dynamics generated.

### Process Overview and Scheduling

The GBABM uses iterated, discrete time steps, each step corresponding to 5 minutes of real time. As per Netlogo convention, each run step is divided into several sub steps.

### Sensing: Role of Quorum Sensing and Implementation in the in vivo GMABM

Expression of virulence genes in *P. aeruginosa *is predominantly controlled by quorum-sensing (QS) regulatory mechanism, a highly conserved "network of networks" regulating hundreds of genes in response to inter-cellular signaling molecules at high population densities [82-84]. While a comprehensive representation of these feedback networks is beyond the scope of GMABM, select components relevant to host-derived cues were included. Although emerging evidence suggests that QS may be less dependent on population density in certain contexts [85], for the purposes of the GMABM, recognition of sufficient local population density by Pseudomonas agents was a pre-condition for virulence activation and expression. The "sufficient" threshold of local Pseudomonas agent population density to trigger the quorum signal is a user-defined initial condition (qualitative scale), while the strength of the virulence expression is augmented by stress-induced host factors. Virulence expression requires both an increase in the simulated bacterial population level beyond set threshold (an initial parameter in the GMABM) and the presence of simulated host stress signals. While in the real world system there is very likely a dynamic interplay between the quorum signal threshold and the mediator milieu for the bacteria, given the current resolution of the GMABM we have chosen to focus on the more direct effects of host stress signaling via adenosine, IFN-γ, dynorphin and phosphate. This is reflected in the Experiments section where the quorum signal threshold was set at a relatively low value, thereby placing focus on the effects of the above noted mediators.

### Interaction

The GMABM is a spatially explicit model, where interactions between agents and their environment are defined by the parcel of discrete space occupied by the agent ("patch" in Netlogo parlance) as well as the Moore neighborhood of that patch, where the Moore neighborhood on a 2-D square grid consists of the 8 squares immediately adjacent to and surrounding the central square. Biological cell-agent to biological cell-agent interactions are generally mediated through the passage of environmental variables produced and sensed by the various agent types; specific cell-to-cell contact interactions (other than adhesion reflected as cessation of Pseudomonas agent movement) are not included in the current development of the GMABM.

### Stochasticity

The existence of stochasticity in intracellular signaling and gene regulation are well accepted [86] and this property is incorporated into the rules for signal transduction, receptor dynamics and gene regulation/expression through the addition of random number modifiers to the likelihood of particular events. The Netlogo software toolkit utilizes the Mersenne Twister as its pseudo-random number generator for its "random" primitives.

### Initialization

There is no dynamic initialization run-period in the GMABM; this means that simulation t = 0 is intended to represent an arbitrary time point in a system that is already at steady state. Baseline simulation conditions represent the reference system in its non-perturbed state, with "normal" levels of bacterial nutrients (including phosphate), fully intact mucus layer, baseline levels of commensal bacteria, GECs with fully intact tight junctions and no active inflammatory mediators. Pseudomonas agents are present, but in the absence of virulence activating cues (see Submodel section below) they do not have their corresponding virulence pathways active. The baseline, non-perturbed state of the GBABM was demonstrated to be stable through as series of non-perturbed simulation runs to 1000 time steps.

### Submodels

This section will describe in detail the underlying biology and the implementation of that biology in the two mobile agent classes: Pseudomonas agents and GEC agents. Pseudomonas agent functions are subdivided into response pathways to specific conditions associated with host stress: ischemia, phosphate depletion, inflammation and opioid presence. The GEC agent functions can be classed into two groups: the first represents the representation of gut barrier function, the primary host function affected by microbial virulence, the second group consists of assignment to GEC agents three of the stress conditions discussed above: ischemia, inflammation and opioid production. In addition, while not a specific agent class, a subsection describing the handling of commensal bacteria as a population-based patch variable is described.

Entity #1: Pseudomonas Agents:

Each virulence activation component was developed with a submodel ABM to allow cross-model validation with their respective experimental referents. Subsequently, the rule sets of these modular submodels were integrated into a single model (the GMABM) intended to be a computational analog to animal models and other more physiologic experimental platforms. Simulated experiments were then performed on the integrated GMABM. Figures 3, 4, 5 and 6 demonstrate schematic representations for each of the four individual virulence pathways represented in the Pseudomonas agent. The aggregated set of pathways present in the GMABM is seen in Figure 7. The following sections will describe each submodel and its associated biology.

• Ischemia: Adenosine-mediated virulence activation

Intestinal ischemia is a contributing factor in the pathogenesis of gut-derived sepsis [9,26]. Intestinal ischemia was simulated by initiating GEC agent expression of its state variable HIF-1α, which initiates production and release of adenosine as an environmental variable. Environmental adenosine present on patches occupied by Pseudomonas agents is converted by adenosine deaminase within the Pseudomonas agents to the internal state variable inosine and initiates the time-scaled expression of cytosolic PA-I lectin (i-PAI-lectin to denote the location of the variable). The time course for peak expression of PA-I lectin in *in vitro *models was in the range of 5-7 hours, and the Pseudomonas agent signal transduction pathway of inosine interaction with the lecA complex was tuned to peak production of i-PAI-lectin at 5 hours. Because expression of PA-I lectin is associated with adhesion to the epithelial cell layer, Pseudomonas agents with positive i-PAI-lectin became fixed to their current patch. Translocation of PA-I lectin to the cell wall was represented as conversion of the agent variable i-PAI-lectin to the patch variable PA-I lectin. The expression and integrity of epithelial tight junctions (occludin) was inversely proportional to patch PA-I lectin concentration, resulting in discrete regions of increased epithelial cell layer permeability around activated microbes. A schematic for this virulence pathway is seen in Figure 3.

• Phosphate depletion, *P. aeruginosa *phosphate sensing and virulence activation

In critical illness and post-surgical stress serum and extra-cellular hypophosphatemia results from phosphatonin-mediated urinary wasting [87,88] and sequestration by vitals organs (heart, brain, etc.). In the ABM, the initial phosphate concentration for each patch was randomly set at given a normal distribution between 0-99 (arbitrary units), but the upper range modifiable through the user interface. The variable state representing the conformational structure of the internal agent variable PstS phosphate-sensing molecule was determined by the phosphate concentration of the current patch. Signal transduction pathways for low phosphate sensing initiated by activation of PstS, including conformational changes in Pst-PhoU-PhoR complex and eventual phosphorylation of transcriptional regulator PhoB were represented as internal agent variables with shared QS components (i.e. MvfR, PQS). A schematic for this pathway can be seen in Figure 4.

• Inflammation: interferon-γ (IFN-γ) activation of multiple virulence pathways

Early recognition of host immune activation could enhance the efficacy and coordination of microbial defense and virulence strategies against host immunity. In *P. aeruginosa*, cytokine-rich media from activated cultured T-cells induces PA-I lectin expression at transcriptional and translational levels [8]. IFN-γ produced by the host is bound to outer membrane porin OprF on Pseudomonas agents. This activates the expression of PA-I lectin. The RhlI, a N-(butanoyl)-L-homoserine lactone synthetase in QS system is activated during exposure to IFN-γ and required for PA-I lectin expression; this suggests a link between OprF and RhII. OprF, RhlI, and PA-I lectin are Pseudomonas agent state variables implemented in a time-scaled pathway to yield peak PA-I lectin expression 6-7 hours following interferon binding, replicating the time course of *in vitro *studies. The current GMABM does not include inflammatory/immune cells; therefore activation of the inflammatory response and subsequent production of IFN-γ is incorporated as a function of GEC agents controlled at the user interface as a simulation experimental condition. A schematic of this virulence pathway is seen in Figure 5.

• Endogenous opioids during host stress and virulence activation

Endogenous opioids are diffusely released during host stress and represent a potential early danger signal for microbes in richly innervated tissues such as the intestinal tract [89-92] and can induce robust, multi-faceted virulence expression in *P. aeruginosa *through activation of key transcriptional regulator MvfR, expression of its regulated operon pqsABCDE and production of downstream signaling molecules HHQ, HQNO, and PQS [26]. HQNO is a potent toxin against gram-positive bacteria including Lactobacillus species, a common representative of endogenous human flora, conferring a competitive advantage for scarce resources in the human gut. PQS, when complexed with scavenged iron and emulsified with secreted rhamnolipids, forms a potent toxic complex that induces apoptosis in intestinal epithelial cell. MvfR, NNQ, NQNO and PQS were represented as Pseudomonas agent state variables in time-scaled, semi-quantitative signal transduction pathways resulting in the three key virulence products. The schematic for dynorphin sensing can be seen in Figure 6. Of particular interest is a putative link between the *pho *box complex and MvfR, which would tie together the pathways for dynorphin and phosphate sensing. This putative interaction is demonstrated in red in the overall schematic for all four virulence pathways seen in Figure 7.

### Movement

Non-adhered Pseudomonas agents move one grid-space per simulation run step in a random fashion; there is no chemotaxis modeled. However, the presence of i-PA-I lectin, produced through pathways for ischemia and inflammation, leads to adhesion of Pseudomonas agents to underlying GEC agents and cessation of movement.

Entity #2: Gut epithelial cells

While the epithelial cell layer primarily governs the reactive surface of the host in the gut milieu, there are notable contributions from various epithelial subtypes (such as goblet cell, which produce mucus) and a host of inflammatory cell subtypes. Given our focus on *P. aeruginosa *virulence activation, we have abstracted and assigned these host functions to the GEC agents as an aggregated proxy for the host component of the gut milieu. The role of gut epithelial cell population behavior as a proxy of host health is represented in their permeability barrier function, reflected as tight junction integrity by the GEC agents.

• Epithelial permeability and tight junction metabolism

The tight junctions are maintained at a steady state though metabolic and localization processes, and these pathways are known to be subject to disruption by inflammatory signals [36,71] and, specifically, the production of PA-I lectin by *P. aeruginosa *[93]. Tight junction failure and subsequent increase in epithelial barrier permeability is a well-recognized sign of gut inflammation and a precondition associated with gut-derived sepsis [36]. Epithelial barrier function can also be compromised by apopotosis, i.e. programmed cell-death. Epithelial apoptosis can be initiated through *P. aeruginosa *produced toxin PQS [6]. GEC agents represent tight junction protein metabolism with dynamic, time scaled turnover of a representative tight junction protein, occludin as a GEC state variable, to its half-life of ~90 minutes (= 18 simulation steps) [94]. Without perturbation to the system, appropriately localized occludin remains at a steady state to maintain tight junction levels and effective epithelial barrier function. The presence of patch PA-I lectin produced by activated Pseudomonas agents interrupts occludin synthesis and results in GEC agent tight junction failure, manifesting as regional loss of barrier function and increases in permeability.

### Simulation control through the epithelial cell agents

User controls on the ABM allowed the possibility of independent simulation of specific aspects of host stress as manifest by the epithelial cell agents (ischemia, inflammation, endogenous opioids and phosphate depletion).

• Intestinal Ischemia

When "Ischemia" is activated via the User Interface the GEC agents produce HIF-1α, which is added as the initiating factor to the production of adenosine by the GEC agents. The adenosine has an intracellular component, which is a state variable for the GEC agents and represents production, and a secreted version, which is released by the GEC agents and diffuses into the environment. The secreted form of adenosine is the environmental patch variable that activates the ischemia signaling rules of the Pseudomonas agents (see above). Furthermore, during the time when "Ischemia" is active the GEC agents decrement their "life" state variable such that they will die if the "Ischemia" persists for 24 hours of simulated time.

• Intestinal Inflammation

As noted above, the GMABM does not include inflammatory cells and the inflammatory response of the gut as an organ is abstracted by the production of IFN-γ by GEC agents. When "Inflammation" is activated (via the User Interface) the GEC agents produce IFN-γ as a diffused environmental variable that activates the inflammation signaling rules of the Pseudomonas agents.

• Endogenous opioid production

When "Stress" or "Ischemia" is activated (via the User Interface) the GEC agents release dynorphin, a representative κ-opioid, as a diffused environmental variable that activates the opioid signaling rules of the Pseudomonas agents.

### Modeling commensal flora as a source of competition for resources

At baseline, both Pseudomonas agents and commensal flora existed within the mucous layer. Commensal flora are represented as patch variables representing aggregate populations of common intestinal bacteria (such as *Lactobacillus *species, *Bacteroides *species). While we recognize that a high degree of diversity exists among commensal gut flora, in the GMABM they were represented collectively as a generic microbial species lacking the genetic background or molecular machinery to express significant virulence towards the host. Because populations of commensal flora are typically several magnitudes greater than those of colonizing microbes, commensal flora was abstractly represented as an environmental spatial variable discretized on the model grid space (as opposed to agents) in order to produce a more realistic scale given computational constraints. Populations of both commensal bacteria and Pseudomonas agents were limited by a finite carrying capacity determined by the volume (thickness) of mucous layer at a specific point in the virtual environment [95]. The growth dynamics of the commensal bacteria are highly abstracted to linear growth with an upper limit based on the mucous-dependent carrying capacity, and their competition limiting the number of non-virulent Pseudomonas agents is manifest by their subtraction of available resource on a particular patch. The mucous layer was modeled as an environmental data layer with properties distinct from the intestinal lumen, particularly in terms of representing available space and nutrients for the simulated bacterial populations. As the carrying capacity of the simulated mucous layer is limited with respect to space and nutrients, Pseudomonas agents have the potential to expand their niche within that environment by eliminating competing commensal flora (i.e. targeted killing of *Lactobacillus*). At the current time, the dynamics of mucous production, sloughing and turnover were not incorporated into the GMABM.

### Cross-model validation of submodel ABMs to biological experimental referents

**Figure 8:**
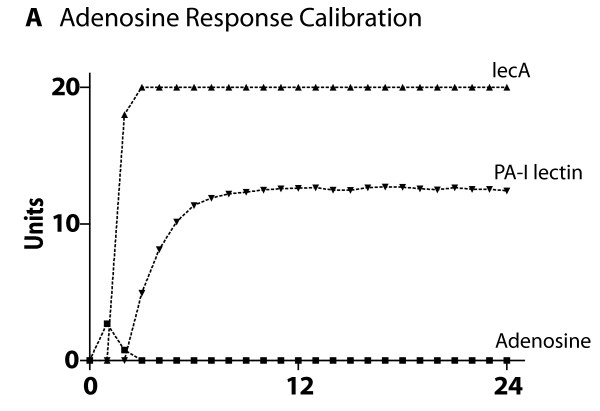
**Cross-model validation of virulence expression in Pseudomonas agents to experimental model of transient ischemia**. This figure demonstrates adenosine-induced Pseudomonas agent expression of *lecA *and subsequent production of PA-I lectin. PA-O lectin would then lead to reduced expression of tight junction proteins in GEC agents (see Additional File 2 for sample experimental referent data).

**Figure 9:**
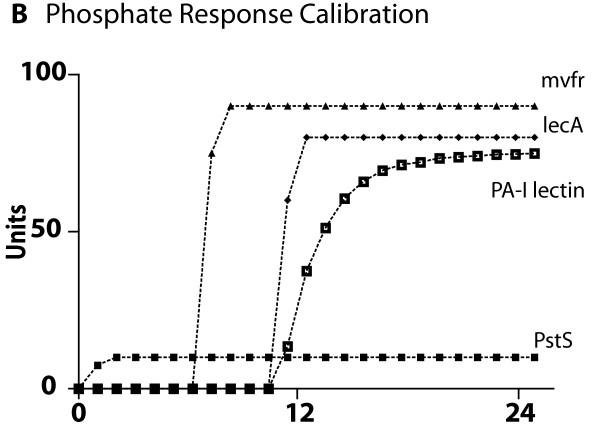
**Cross-model validation of virulence expression in Pseudomonas agents to experimental model of low phosphate**. These simulations of low phosphate conditions show the results of Pseudomonas agent virulence activation in response to low phosphate sensing, reflected in the production of PstS, MvfR, *lecA *and PA-I lectin (see Additional File 3 for sample experimental referent data).

**Figure 10:**
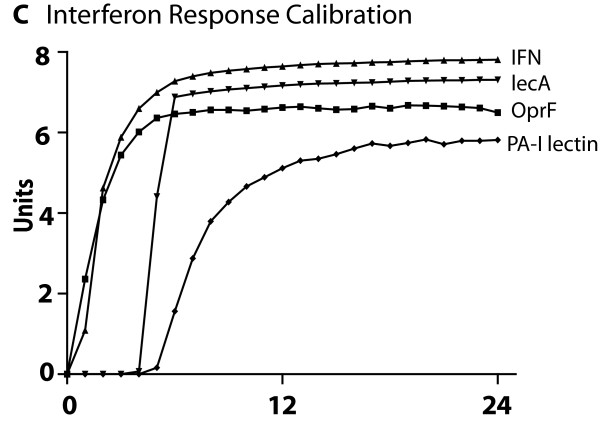
**Cross-model validation of virulence expression in Pseudomonas agents to experiments of gut epithelial immune activation**. This figure displays the results of simulations of GEC agent production of IFN-γ with binding to Pseudomonas agent surface receptor OprF, and subsequent Pseudomonas agent production of PA-I lectin (see Additional File 4 for experimental referent data).

**Figure 11:**
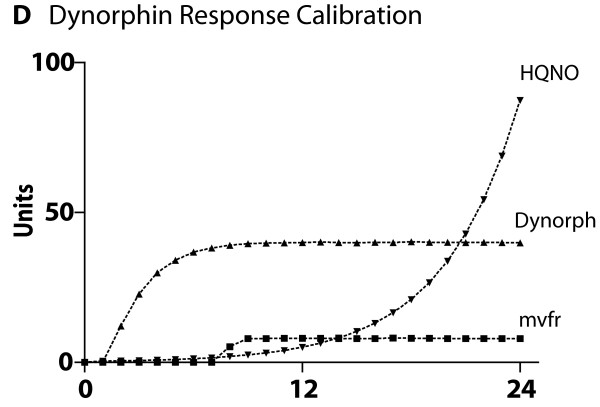
**Cross-model validation of virulence expression in Pseudomonas agents to experiments of endogenous opioid production**. This figure demonstrates the results of simulations of the production of endogenous dynorphin by GEC agents in response to a simulation of 20 minutes of ischemia, and the effects of the dynorphin production on Pseudomonas agents' levels of MvfR and HQNO production (see Additional File 5 for sample experimental referent data). Note that there is a discrepancy in the final trajectory of HQNO production between the ABM and the experimental referent. However, the effect of this discrepancy is not apparent in the following figures that demonstrate the suppression of commensal bacterial growth.

**Figure 12:**
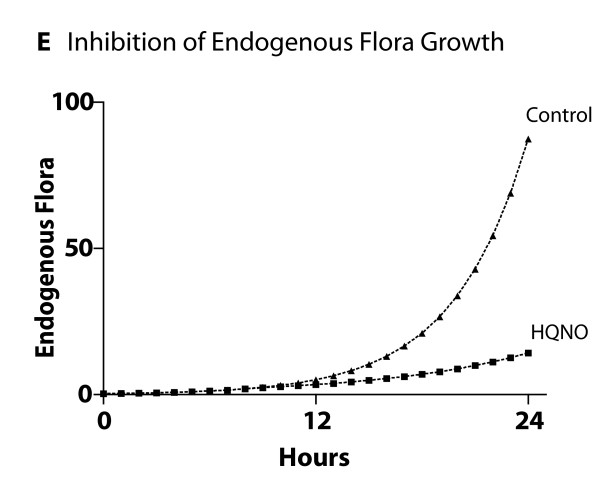
**Cross-model validation of virulence expression in Pseudomonas agents in experiments of endogenous opioid production manifesting as suppression of commensal bacterial populations**. This figure demonstrates the results of simulations of the production of endogenous dynorphin by GEC agents in response to a simulation of 20 minutes of ischemia, activation of the virulence factor HQNO in the Pseudomonas agents and its effect on the suppression of the growth of commensal bacteria (compare to Additional File 6).

**Figure 13:**
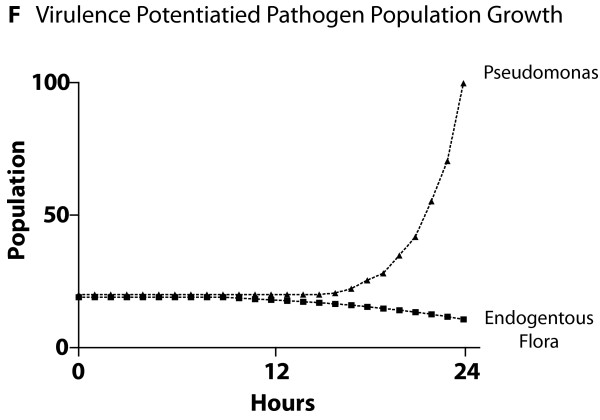
**Cross-model validation to experiments of endogenous opioid production concerning the population dynamics of Pseudomonas agents and endogenous flora**. The competitive advantage of the Pseudomonas agents is due to the suppression of commensal bacteria resulting from the Pseudomonas agents' activation of virulence factors and production of HQNO (which inhibits commensal bacterial growth) (information extracted from Additional File 6).

The ABMs for each of the four central host-derived signals for virulence expression in *P. aeruginosa *were cross-model validated to their respective experimental referents prior to their integration into the GMABM, which was then used to perform simulated experiments to examine the system level consequences of stress on the gut ecosystem. The summary results of these simulations can be seen in Figures 8, 9, 10, 11, 12 and 13. Representative data figures from the referent publications can be seen in Additional Files 2, 3, 4, 5 and 6.

• Effects of adenosine resulting from ischemia/reperfusion

Ischemic conditions simulating occlusion of mesenteric vessels were established by adjusting control settings on the user interface that initiated production of HIF-α and release of adenosine by GEC agents to match the published data in Patel et al [9] (representative figure seen in Additional File 2). The relatively short half-life, rapid diffusion and uptake by Pseudomonas agents were reflected in the diffusion and degradation parameters in the GMABM. Simulated transient ischemia/reperfusion (30 minutes) yielded peak PA-I lectin expression 7 hours after activation with time-calibrated events to account for absorption and enzymatic conversion to inosine, a potent activator of *lecA *promoter for PA-I lectin (Figure 8).

• Effects of hypophosphatemia

*P. aeruginosa *is sensitive to ambient phosphate concentrations and responds to phosphate depletion by expressing a robust virulent phenotype. The model output behavior of these pathways as implemented in Pseudomonas agents was fitted to the data published in Long et al [23]. Note that the representative figure in Additional File 3 is a lethality curve that represents the time course of the activity of PstS and PA-I lectin discussed within that paper. The phosphate depletion induces the expression of the Pseudomonas agent phosphate sensor PstS; interacting with the same QS circuitry as other host-derived cues, low phosphate concentration leads to peak MvfR activation at 7 hours and PA-I lectin expression at 10 hours with plateaus present until 24 hours (Figure 9).

• Effects of interferon-γ due to immune activation

*P. aeruginosa *recognizes host immune activation through binding of interferon-ϒ to membrane-bound OprF receptor and interaction with the QS circuitry. The model output behavior of these pathways as implemented in Pseudomonas agents was fitted to the data published in Wu et al [8] (see representative figure in Additional File 4). With similar signal transduction architecture to other sense and response mechanisms, Pseudomonas agent peak PA-I lectin expression follows IFN-γ concentrations and peaks at 7 hours (Figure 10).

• Effects of endogenous opioids

The model output behavior of the effects of dynorphin as implemented in Pseudomonas agents was fitted to the data published in Zaborina et al [26] (see representative data figures reproduced as Additional Files 5 and 6). The effects of dynorphin on Pseudomonas agents demonstrated the activation of the quorum-sensing (QS) control element MvfR resulting in peak expression 10 hours after activation (Figure 11). We note that there is a discrepancy between the apparent trajectory of HQNO production between the ABM seen in Figure 11 and 11the reference data (Additional File 5). However, as the production of HQNO by activated Pseudomonas agents decreases the growth rate of commensal microbial flora (i.e. *Lactobaccillus *species), the discrepancy in HQNO trajectory is accounted for by the inhibitory effect of HQNO on commensal flora growth in the presence of dynorphin based on corresponding *in vitro *studies [26] and seen in Figure 12 (compare to growth inhibition seen in Additional File 6). Additionally, given the fact that the GMABM has a fixed nutrient carrying capacity, the reduction in competition for resources associated with decreased commensal flora growth provides an opportunity for corresponding Pseudomonas agent population growth (Figure 13).

### Overall Model: Integration of modular ABM components to simulate gut-microbe interactions in a stressed environment

The modular submodel rule sets were integrated into a single "*in vivo*" GMABM intended to be a computational analog to animal models and other more physiologic experimental platforms. The behavioral algorithms for sense and response virulence regulation were integrated in Pseudomonas agents maintaining the time-scaled and semi-quantitative properties of signal transduction pathways from the in vitro ABMs. The dynorphin and phosphate sensing submodels connected through the *pho *box-MvfR interaction; the interferon, dynorphin and adenosine signaling pathways all converge on lecA. Subsequent simulation experiments were performed using the integrated GMABM.

### Determination of initial Pseudomonas agent populations

**Figure 14:**
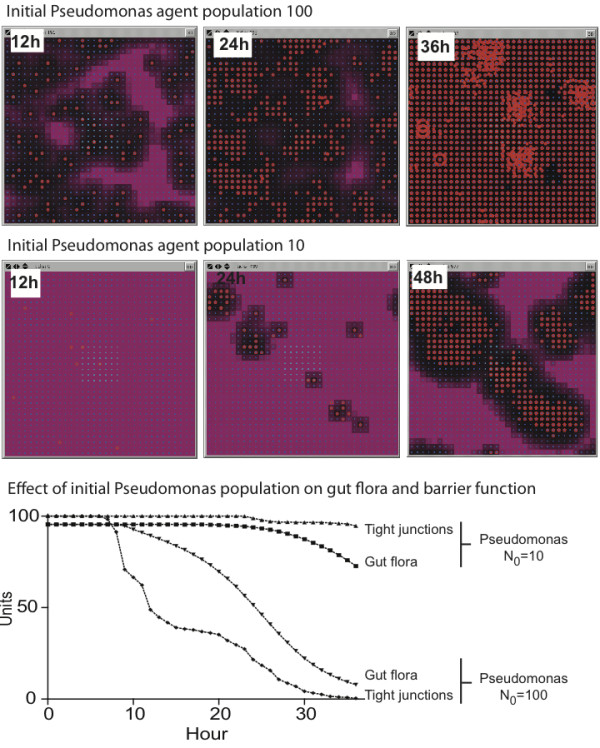
**Effect of initial *Pseudomonas *agent population on simulated host injury**. Selected frames from the model interface during simulation of phosphate depletion depict *Pseudomonas agents *(red pentagons) and tight junctions (purple background), with black background indicating severe barrier disruption. Upper Row of Screenshots: An initial *Pseudomonas *agent population of 100 agents produced rapid and severe barrier disruption within 12 hours of phosphate depletion and near complete at 36 hours. We considered this to a disproportionally lethal response and non-realistic calibration behavior. Second Row of Screenshots: An initial population of 10 Pseudomonas agents produced moderate injury after 48 hours of phosphate depletion. These dynamics appeared to meet the standard of face validity with respect to the clinically relevant situation, and provided an enhanced ability to identify the properties of the system's tipping point. Graph: These graph demonstrates the relative effect on commensal bacteria and GEC tight junctions at N_0 _= 100 and 10 respectively.

For parameter estimation in the simulated *in vivo *experiments, an initial Pseudomonas agent population = 100 was arbitrarily chosen as the start point for the tuning of virulence gene expression. GMABM simulation runs with initial Pseudomonas agents = 100 produced severe defects in barrier function and marked reduction in commensal flora populations as early as 12 hours after insult, with very high levels of toxin production (HQNO), rapid decrease in commensal flora populations and very rapid Pseudomonas agent colony growth (Figure 14, uppermost row of screenshots, and Pseudomonas N_0 _= 100 on graph). In our assessment, this appeared to be too severe an effect, not reflective the state space of the clinically relevant situation, and would not allow investigation of the critical dynamics of the "tipping point" of the system. Therefore, the initial Pseudomonas agent population was reduced to produce a more qualitatively realistic progression of injury. This was accomplished with an initial Pseudomonas agent number = 10 (Figure 14, second row of screenshots, and Pseudomonas N_0 _= 10 on graph). By producing a more realistic injury progression this initial agent level appeared appropriate for the in vivo GMABM's level of resolution, allowing clearer demonstration of the spatial and temporal heterogeneity of the gut environment. Given this tuning, the GMABM demonstrated expression of PA-I lectin and bacterial adhesion appearing at 12 hours post transient ischemic insult, followed by mild disruption of barrier function at 36 hours and then severe regional defect at 48 hours. Similarly, simulated Pseudomonas agent population growth emerged at 36 hours with contiguous colony expansion and barrier disruption at 48 hours (Figure 14, graph). These results do not have specific *in vitro *experiments as their reference points; rather, these dynamics are assessed using the standard of face validity discussed above [68,69] and used to identify initial simulation conditions sufficient to generate behaviors correlating to those of experimental and clinical interest reflected in the simulation experiments below.

## Results

There are three primary goals of the GMABM simulation experiments:

(a) Investigate potential experimental conditions not yet developed.

(b) Examine the sufficiency of existing hypotheses to explain the experimental data.

(c) Potentially guide future experimental design.

In terms of (a); we present a simulated experiment of non-lethal intestinal ischemia/reperfusion, a circumstance very clinically relevant and seen in conditions associated with the later development of gut-derived sepsis, such as hemorrhagic shock, abdominal aortic surgery and initial septic shock of non-gut origin. We use the results of these simulated experiments to fulfill goal (c) and suggest periods of interesting dynamics that might affect the timing of sample acquisition in future laboratory experiments. In terms of (b), we examine a divergence between the simulation output of the GMABM and referent experimental data, and use that insight to effect goal (c) and posit additional factors that might be investigated to afford reconciliation, some of which have been pursued.

### Simulating non-lethal transient intestinal ischemia

**Figure 15:**
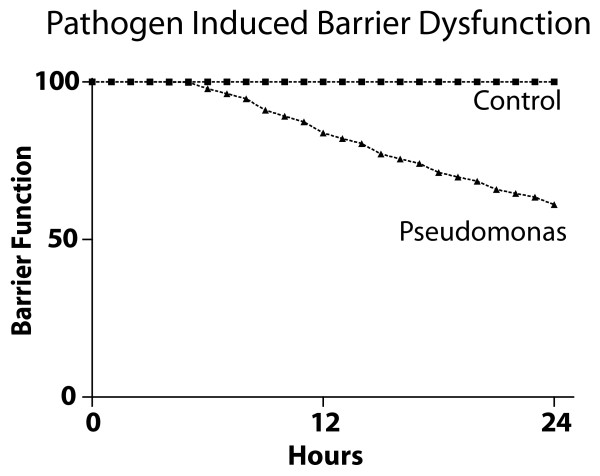
**GMABM response to transient intestinal ischemia**. The effects of 30 minutes of transient intestinal ischemia, including release of dynorphin and adenosine into the intestinal lumen, were simulated. We utilized two *in silico *experimental groups: a control group (Pseudomonas agents = 0) and a Pseudomonas group (Pseudomonas agents = 10). Transient ischemia alone produced no significant disruption (Control). The combination of simulated transient intestinal ischemia and the presence of Pseudomonas agents yielded a 40% decrease in barrier function at 24 hours of simulation time. Note that the initiation of the effect can be seen between 6 and 12 hours, suggesting that this period should be targeted for sampling in any future *in vivo *experiments to obtain the most potentially relevant data.

In the traditional laboratory setting, effluent from the lumen of intestinal segments following ischemia reperfusion injury was used in *in vitro *experiments to study the effects of endogenous opioids and ischemia byproducts on virulence expression in *P. aeruginosa *[9,26]. The GMABM was used to simulate 30 minutes of segmental intestinal ischemia followed by reperfusion injury. Immune activation and phosphate depletion were initially excluded from the execution of the ischemia/reperfusion simulation. Presently, there are no published data evaluating *Pseudomonas *virulence expression and host survival using an animal model of non-lethal transient intestinal ischemia. However, the GMABM simulations demonstrated that exposure to select microenvironment changes in the gut subsequent to transient ischemia/reperfusion injury produced significant barrier dysfunction (Figure 15), setting the stage for potential host morbidity. This condition has clinical relevance, as often the onset of critical illness and systemic inflammation is associated with transient hypotension and hypoperfusion prior to onset of resuscitative measures [36]. This simulation data also suggests that an *in vivo *experiment to confirm this type of behavior should focus its sample collection in the 6-12 hour time frame post -ischemia, and that higher frequency measurements in this time frame would be substantially more informative as opposed to collections beyond the 12-hour time point. Work is currently ongoing in terms of the design of the appropriate *in vivo *model in which to evaluate non-lethal transient intestinal ischemia with a focus on the appropriate phenotypic metrics and high-throughput analysis (metagenomics and metatrascriptomics) with which to separate groups.

### Reconciliation with in vivo models of gut-derived sepsis

We have developed and published a reproducible model of gut-derived sepsis in mice, where mice were starved preoperatively, underwent laparotomy with 30% hepatectomy, followed by cecal injection of *P. aeruginosa *[5]. Mice exposed to hepatectomy alone or starvation alone both have very low morbidity and mortality, suggesting that each component contributes to a specific and necessary stress signature at the host-microbe interface. Partial hepatectomy produces systemic physiologic changes related to surgical stress such as immune activation, catabolic state and phosphate depletion in the intestinal mucous layer. The molecular details of the stress signature in the intestinal host-microbe interface are unclear, but may include immune mediators such as IFN-γ, which is known to induce virulence expression in *P. aeruginosa*. Starvation prevents the repletion of phosphate in the mucous layer in addition to the systemic effects of nutritional depletion. The requirement of two stress events to produce morbidity and mortality as a consequence of virulence expression has also been observed in a wet lab model of *C. elegans*, where the addition of starvation or heat stress was required to potentiate the effect of phosphate depletion [24]. The reason for this is unclear, but likely involves effects on host defenses as well as microbe virulence expression.

**Figure 16:**
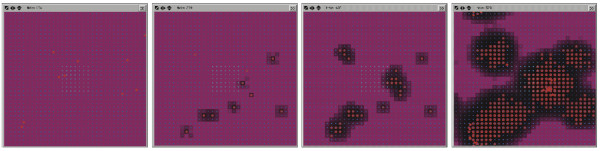
**GMABM response to phosphate depletion alone**. Selected frames from the model interface taken at 6, 12, 24 and 48 hrs of simulated time during simulation of phosphate depletion in the integrated GMABM. Pseudomonas agents are red pentagons and intact tight junctions are seen as the purple background, with black background indicating severe barrier disruption. Phosphate depletion alone yielded moderate to severe barrier disruption after 48 hours. This response was more severe than expected given prior experimental referent data.

**Figure 17:**
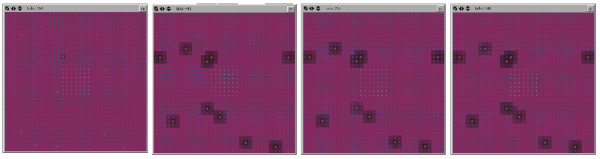
**GMABM response to host stress without phosphate depletion**. Selected frames from the model interface taken at 6, 12, 24 and 48 hrs of simulated time during simulation of systemic inflammation (IFN-ϒ) without phosphate depletion in the integrated GMABM. Pseudomonas agents are red pentagons and intact tight junctions are seen as the purple background. This set of simulations demonstrate only mild barrier disruption (grey-black areas), consistent with experimental findings that immune activation alone in *in vivo *models is not sufficient to generate a clinically significant injury.

**Figure 18:**
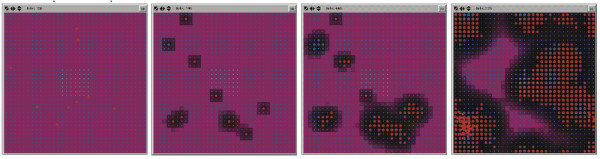
**GMABM response to host stress and phosphate depletion**. Selected frames from the model interface taken at 6, 12, 24 and 48 hrs of simulated time during simulation of systemic inflammation (IFN-ϒ) with phosphate depletion. Pseudomonas agents are red pentagons and intact tight junctions are seen as the purple background, with black background indicating severe barrier disruption. Phosphate depletion in conjunction with inflammation combination resulted in the most severe disruption of barrier function. This finding is consistent with that suggested in prior experimental studies.

**Figure 19:**
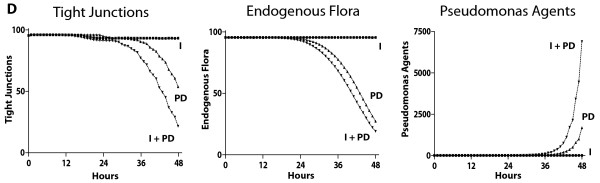
**GMABM response to host stress and low phosphate with respect to Pseudomonas agent and commensal flora population dynamics**. The combination of phosphate depletion and inflammation produced the greatest reduction in endogenous flora and increase in *Pseudomonas *agent population over the course of 48 hours simulated time.

The GMABM was used to simulate hepatectomy with phosphate repletion (inflammation alone), starvation (phosphate depletion alone) and the combination of the two (inflammation and phosphate depletion). Simulation of phosphate depletion alone induced a moderate to severe intestinal barrier dysfunction and reduction of commensal gut flora by 48 hours, with the first indications of patchy breakdown between 24 and 36 hours (Figure 16). Alternatively, simulation of systemic inflammation without phosphate depletion produced mild, localized patches of barrier defect without progression from 24 to 48 hours, corroborating the *in vivo *results suggesting immune activation alone is insufficient to generate a clinically significant injury (Figure 17). Simulation of the combined effects of phosphate depletion and inflammation yielded a more severe injury than phosphate alone, most evident at the 48 hour time point with increased Pseudomonas agent population growth resulting in more severe disruption of barrier function and greater reduction in commensal flora (Figures 18 and 19).

The results from *in vivo *studies in the animal model of gut sepsis largely correlate with the behavior observed in the GMABM, with one exception. In the laboratory, starvation induces depletion of phosphate in intestinal mucous, but does not alone lead to morbidity in the animal following cecal injection of *P. aeruginosa *[23]. The GMABM predicts a more severe injury than expected in response to only phosphate depletion (Figure 16). This corresponds to hepatectomy without starvation in the GMABM, a low mortality condition in the biological experiments. We view this discrepancy as representative of exactly the case of a "broken model" that is necessary to advance knowledge; given the explicit nature of the GMABM its inability in this particular context to produce output matching the biological referent provides insight into what additional modifications and investigations need to be pursued in more detail in the next cycle of experiment and modeling. These future investigations will be elaborated upon in the Discussion.

## Discussion

Biology is currently a science in its the discovery phase, where incomplete knowledge, ambiguity and uncertainty are inevitably present. We suggest that the detailed, selectively qualitative approach to dynamic knowledge representation lies in the fertile middle ground between high resolution, quantitative predictive models and highly abstracted models of generalize-able functions. This range as previously been well displayed in Hunt, et al [34]. It also serves as a reminder that the process of modeling involves selective abstraction with the goal of gaining insight into the system being modeled.

Expanding on the role of dynamic knowledge representation, a further advantage of *in silico *experimental environments is the ability to implement experimental conditions not yet developed. This can be useful in the future planning of lines of investigation, and can potentially predict behaviors when those experimental preparations are eventually developed [96]. An example of this capability is demonstrated in the simulations of non-lethal transient intestinal ischemia, a condition more likely to be clinically relevant than the extreme ischemic insults generally present in wet lab models. As noted above, while there is currently no published *in vivo *model of virulence activation in non-lethal intestinal ischemia/reperfusion injury, the GMABM simulations suggest a time frame of interest for future sample collections when those laboratory models are developed. This capability may also be used to refine sampling intervals in existing wet lab models by uncovering unanticipated dynamic shifts within current sampling periods.

The behaviors observed in the GMABM during simulated major surgical stress correlated with existing animal model findings, with only one exception: the GMABM demonstrated substantial barrier defect in the context of mucosal phosphate depletion alone, while in the animal model, phosphate depletion associated with starvation alone did not produce significant morbidity or mortality, but required a second stress signal (hepatectomy). As noted above, we view this discrepancy as an opportunity to investigate additional significant factors involved in the *in vivo *manifestation of *P. aeruginosa *virulence effects. We make a series of initial hypotheses concerning the source of discrepancy between the *in vivo *experiments and the GMABM. These include:

1. Exacerbation of the importance of phosphate sensing on *P. aeruginosa *virulence effects. The phosphate sensing pathways are explicitly modeled in the Pseudomonas agents, but without potential corresponding control pathways that may result in protective redundancies that provide a damping effect on the low-phosphate sensing triggers of virulence. In order to investigate additional control systems affecting the low phosphate response, we have already initiated a series of experiments concerning the complementary importance of iron sensing and metabolism with respect to low-phosphate induced virulence (manuscript in preparation). Also, as noted above, there is also a suggestion of a potential link between the phosphate sensing pathway and dynorphin response pathway (demonstrated in Figure 7 in terms of a putative link between *pho *box and MvfR). Negative feedback or concurrent pathway dependencies may result in synergistic damping of the virulence response if this link were established. We are also in the process of investigating this relationship with additional experiments.

2. Under-representation of the dynamics of quorum sensing and its influence on *P. aeruginosa *population dynamics. The pathways of virulence expression related to phosphate depletion are dependent on interactions with the quorum sensing system, a highly conserved hierarchical circuitry, but as noted in the Methods section, the GMABM does not incorporate a dynamic switch that links phosphate sensing to quorum sensing. This relationship has been demonstrated [25], but was not incorporated into the current ABM given the modeling goal of focusing on the specific virulence pathways. However, given the identified discrepancy between the GMABM and the experimental findings, we can posit that by setting the quorum-sensing threshold at the lower point and not accounting for its dynamic shift resulting from low phosphate sensing that the GMABM accelerated and exaggerated the virulence due to low phosphate. This will be rectified in future versions of the GMABM that will incorporate a more comprehensive representation of the quorum sensing modules and its controls. It is also the case that details of those interactions derived from *in vitro *studies may be incomplete, suggesting that the presence of signaling mediators from other stress signals may be required for complete activation and expression of virulence products. This is further supported by observations in *C. elegans *models for assessing *P. aeruginosa *virulence where heat stress or starvation is required to potentiate the effects of phosphate depletion [24].

3. Host physiology may be altered in important ways with the addition of a second stressor that are not represented in the current GMABM. These alterations may include accentuating the catabolic state and phosphate sequestration, enhancing immune activation, altering intestinal mucous production and other components of gut barrier defenses. Given the bacteriocentric emphasis of the current GMABM, these host systemic factors are highly abstracted, and therefore may (and almost certainly does) miss key host dynamics that influence *P. aeruginosa *virulence effects. We note, however, that this discrepancy *would have been present in any standard progression of experimental models! *The benefit of the use of agent-based modeling as dynamic knowledge representation is that it represents a formal and explicit instantiation of the particular hypothesis, and therefore can provide more directed guidance as to that hypothesis' limitations and means to address them in the next cycle of experiments and modeling.

Resolving discrepancies between ABM and animal model behaviors may require consideration and representation of both the host and microbe in greater detail. However, as evidenced in the recent history of translational biomedical research, the range of possible details to be expanded upon may be too great to be evaluated in an efficient, or even tractable, manner [70]. *In silico *dynamic knowledge representation provides an opportunity for the research to engage in "thought experiments" about plausible lines of investigation, reducing the intellectual and pragmatic overhead in exploring hypothesis-space, thereby enhancing the throughput of hypothesis evaluation. Of course, the direction of the thought experiments would be informed by the researcher's expertise and intuition. For instance, in the case of the GMABM:

1. Investigation into mucous barrier physiology and immune activation may provide justification and guidance for future modifications of the GMABM to test hypotheses about their role in determining extent of host injury. As noted above, the current GMABM highly abstracts the host functions concerning the dynamics of maintaining the mucus layer and, particularly, the immune response. Researchers with a particular interest and expertise in these areas could speculate as to how their mechanisms of interest would affect the gut HPI. Dynamic knowledge representation would allow them to implement much more detailed instantiations of these mechanisms *as well as alternative hypotheses *in the GMABM. The behavior of the ABM may then suggest the type and design of experiments to test and validate these hypotheses, forming part of an iterative loop of observation, analysis, synthesis and testing.

2. A more detailed representation of the bacterial ecology in the gut, including metabolism, selection, fitness and succession, could result in the incorporation of more detailed bacterial models into an ABM framework. For instance, there is extensive work in the area of bacterial metabolism, extending to genome scale models of complete bacterial metabolic networks [97-101]; there may be some point at which this degree of detailed representation will provide insight into the ecological dynamics associated with virulence activation. Currently, the ability to link dynamic models of signaling (essentially mandating a change of state) and flux-balance based models of metabolism (predicated on steady-state assumptions) is an open area of investigation. However, the modular architecture of an ABM offers the capability to add and integrate models of such increased detail as the science advances.

The expansibility of ABMs as means of dynamic knowledge representation would allow the consolidation of multiple streams of mechanistic research into aggregated ABMs to simulate increasingly complex biological systems. For instance, in the integrated GMABM the behavior of Pseudomonas agents reflects the combined, simultaneous effects of all four of the central sense and response pathways for virulence regulation in response to host stress. We propose that the GMABM of the HPI in an abstracted model of gut derived sepsis is a starting point to advance the application of dynamic knowledge representation to the translational efforts regarding the role of the gut HPI and its relationship in the pathogenesis of nosocomial infections. Furthermore, we suggest that there is a beneficial role for the use of such relatively abstract and qualitative models in the current research environment. In particular, given the inevitable and unavoidable gaps in knowledge, the use of this type of dynamic knowledge representation as a "virtual sandbox" where researchers can try out new ideas, construct novel hypotheses and instantiate ambiguous or competing mechanisms, will be a necessary capability for dealing with an increasingly data-rich and high-throughput world.

## Competing interests

The authors declare that they have no competing interests.

## Authors' contributions

JS and GA conceived of the project, developed and used the computational model and provided most of the text. OZ and JA provided biological and microbiological background, laboratory experimental results and edited the text. All authors have read and approved the final manuscript.

## Supplementary Material

Additional file 1 **Text File of Netlogo code for the Gut Milieu ABM**. The entities, variables and rules are listed in this file; the components of the user interface are seen at the end of the file. Interested parties may download the actual model from http://bionetgen.org/SCAI-wiki/index.php/Main_Page. Click here for file

Additional file 2 **Representative data file from original paper concerning the effect of hypoxia and adenosine demonstrates the production of the PA-I lectin by *P. aeruginosa *when exposed to media from control, gut epithelial cells over-expressing HIF, and gut epithelial cells exposed to hypoxia (Figure 2C from the original publication)**. Reproduced with permission from [9]. Click here for file

Additional file 3 **Representative data file from original paper concerning the effect of low phosphate is a 48 hr Kaplan-Meier survival curve for mice undergoing major surgery (hepatectomy) (Figure 3A from the original publication), where phosphate is known to be depleted and lethality has been identified to be due to *P. aeruginosa *production of PA-I lectin (details in the text)**. Reprinted with permission from [23]. Click here for file

Additional file 4 **Representative data file from original paper on the effect of IFN-γ demonstrates the response of PA-I lectin promoter activity in *P. aeruginosa *exposed to various pro- and anti-inflammatory cytokines (Figure 1B from the original publication)**. Note that the only active curve is that associated with IFN-γ. Reprinted with permission from [8]. Click here for file

Additional file 5 **Representative data file from original paper on opioid effects demonstrates the production of HQNO in response to the synthetic opioid dynorphin (Figure 2C, panel 3 from the original publication)**. We note that there is a discrepancy in what appears to be the final trajectory of HQNO production between the ABM (rising) and this figure (plateau or slightly decreasing). However, this discrepancy does not appear to influence the effect of HQNO in inhibiting the growth of commensal bacteria (see Additional File 6). Additional File 5 is reprinted with from [26] under the Creative Commons License. Click here for file

Additional file 6 **Representative data file from original paper on opioid effects demonstrates the role of HQNO in inhibiting commensal bacterial growth as seen in the difference between the filled triangles and the other two plots (Figure 7D from the original publication)**. Additional File 6 is reprinted with from [26] under the Creative Commons License. Click here for file
