# Supplementary figures and images for: Agent-based dynamic knowledge representation of Pseudomonas aeruginosa virulence activation in the stressed gut: Towards characterizing host-pathogen interactions in gut-derived sepsis
Source: Theor Biol Med Model. 2011 Sep 19;8:33. doi: 10.1186/1742-4682-8-33 (PMC3184268; doi:10.1186/1742-4682-8-33)

**C**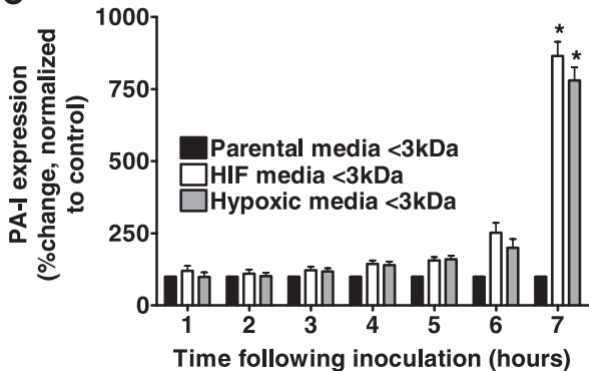

Supplement: Additional file 2 — Representative data file from original paper concerning the effect of hypoxia and adenosine demonstrates the production of the PA-I lectin by P. aeruginosa when exposed to media from control, gut epithelial cells over-expressing HIF, and gut epithelial cells exposed to hypoxia (Figure 2C from the original publication). Reproduced with permission from [9]. [file 1742-4682-8-33-S2.PDF]

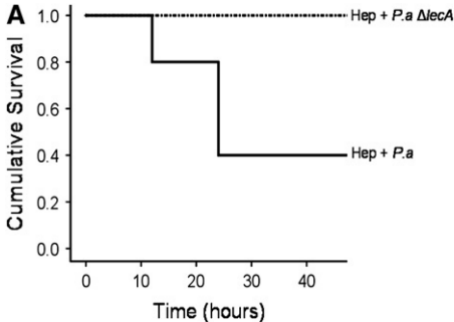

Supplement: Additional file 3 — Representative data file from original paper concerning the effect of low phosphate is a 48 hr Kaplan-Meier survival curve for mice undergoing major surgery (hepatectomy) (Figure 3A from the original publication), where phosphate is known to be depleted and lethality has been identified to be due to P. aeruginosa production of PA-I lectin (details in the text). Reprinted with permission from [23]. [file 1742-4682-8-33-S3.PDF]

**B**Relative PA-I  
promoter activity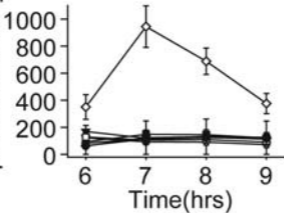

- IL-2
- IL-4
- IL-6
- IL-8
- IL-10
- IL-12
- TNF- $\alpha$
- IFN- $\gamma$

Supplement: Additional file 4 — Representative data file from original paper on the effect of IFN-γ demonstrates the response of PA-I lectin promoter activity in P. aeruginosa exposed to various pro- and anti-inflammatory cytokines (Figure 1B from the original publication). Note that the only active curve is that associated with IFN-γ. Reprinted with permission from [8]. [file 1742-4682-8-33-S4.PDF]

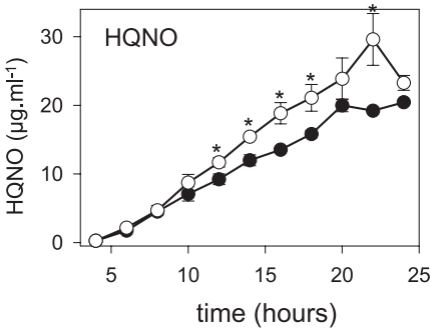

Supplement: Additional file 5 — Representative data file from original paper on opioid effects demonstrates the production of HQNO in response to the synthetic opioid dynorphin (Figure 2C, panel 3 from the original publication). We note that there is a discrepancy in what appears to be the final trajectory of HQNO production between the ABM (rising) and this figure (plateau or slightly decreasing). However, this discrepancy does not appear to influence the effect of HQNO in inhibiting the growth of commensal bacteria (see Additional File 6). Additional File 5 is reprinted with from [26] under the Creative Commons License. [file 1742-4682-8-33-S5.PDF]

**D**

growth (OD 600 nm)

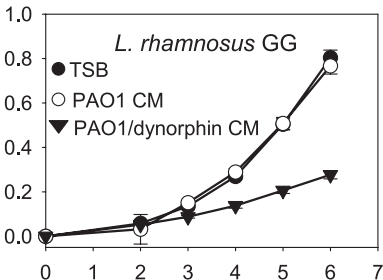

Supplement: Additional file 6 — Representative data file from original paper on opioid effects demonstrates the role of HQNO in inhibiting commensal bacterial growth as seen in the difference between the filled triangles and the other two plots (Figure 7D from the original publication). Additional File 6 is reprinted with from [26] under the Creative Commons License. [file 1742-4682-8-33-S6.PDF]
